# Report of Deaths in the City of Buffalo for the Month of March, 1862

**Published:** 1862-05

**Authors:** Sandford Eastman

**Affiliations:** Health Physician


*Report of Deaths in the City of Buffalo for the month of March*, 1862.

Abscess. 1; Accident, 2; Anæmia, 1; Aneurism, 1; Apoplexy, Cerebral, 4; Asthma, 1; Brain, softening of, 1; Bronchitis, 1; Cirrhosis of Liver, 1; Consumption, 17; Convulsions, 13; Croup, 1; Dentition, 1; Diarrhœa, 2; Disease of the Brain, 2; Disease of the Heart, 4; Disease of the Liver, 2; Disease of the Lungs, 1; Diphtheria, 6; Dropsy, 2; Dropsy, Abdominal 1; Emphysema, 1; Erysipelas, 1; Fever, 1; Fever, Puerperal 2; Fever, Scarlet, 9; Fever, Typhoid, 3; Fever, Typhus 1; Gangrene, 1; Hernia, 1; Inflammation of Bowels, 2; Inflammation of Brain, 11; Inflammation of Brain and Meninges, 1; Inflammation of Liver, 1, Inflammation of Lungs, 8; Inflammation of Lungs, Typhoid, 2; Inflammation of Peritoneum, 1; Inflammation of Womb, 1; Intemperance, 2; Kidneys, Bright's Dis. of, 1; Marasmus, 1; Measles, 1; Old Age, 6; Paralysis, 3; Pemphigus, 1; Pyæmia, 1; Rheumatism, 1; Scrofula, 1; Unknown, 7; —Deaths from Disease, 137; Still born, 5;—Total 142.

SANDFORD EASTMAN, M. D. *Health Physician.*

